# Supplementary material for: Supported Imidazolium-Based Ionic Liquids on a Polysulfone Matrix for Enhanced CO2 Capture
Source: Polymers (Basel). 2022 Nov 11;14(22):4865. doi: 10.3390/polym14224865 (PMC9698076; doi:10.3390/polym14224865)
Supplement: Supplementary file 1 [file polymers-14-04865-s001.zip › polymers-2013953-supplementary.pdf]

polymers MDPI

Supplementary Information: Supported imidazolium-based ionic liquids on polysulfone matrix for enhanced CO_2 capture

David Domingo Huguet [1,2], Aitor Gual [1], Ricard Garcia-Valls[1,3] and Adrianna Nogalska [1,*]

[1] Eurecat, Centre Tecnològic de Catalunya, Unitat de Tecnologia Química, C/Marcel·lí Domingo, 2, 43007 Tarragona, Spain;

[2] Faculty of Chemistry, Universitat Rovira I Virgili, C/ Marcel·lí Domingo 1, 43007 Tarragona, Spain

[3] Department of Chemical Engineering, Universitat Rovira I Virgili, Av. Països Catalans, 26, 43007 Tarragona, Spain

* Correspondence: adrianna.nogalska@eurecat.org (A.N.); Tel.: +34-977-297-089

1. Set-up

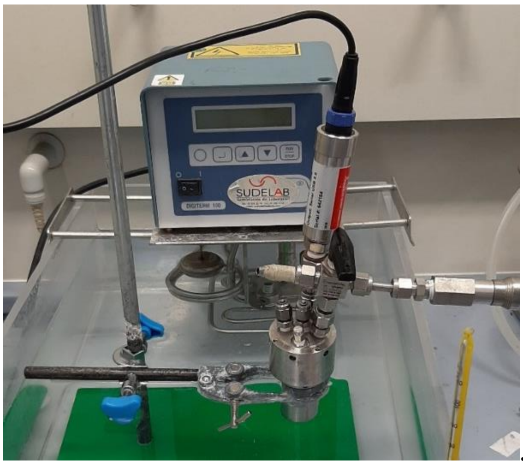

Figure S1. Real set-up for solubility experiments.

2. Ionic liquids characterization:

2.1. General elemental characterization

2.1.1. Compound 1: BMI.Cl

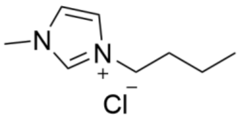

1-butyl-3-methylimidazolium chloride
Chemical Formula: $C_8H_{15}ClN_2$
Exact Mass: 174,09
Molecular Weight: 174,67
m/z: 174.09 (100.0%), 176.09 (32.0%), 175.10 (8.8%), 177.09 (3.0%)
Elemental Analysis: C, 55.01; H, 8.66; Cl, 20.30; N, 16.04

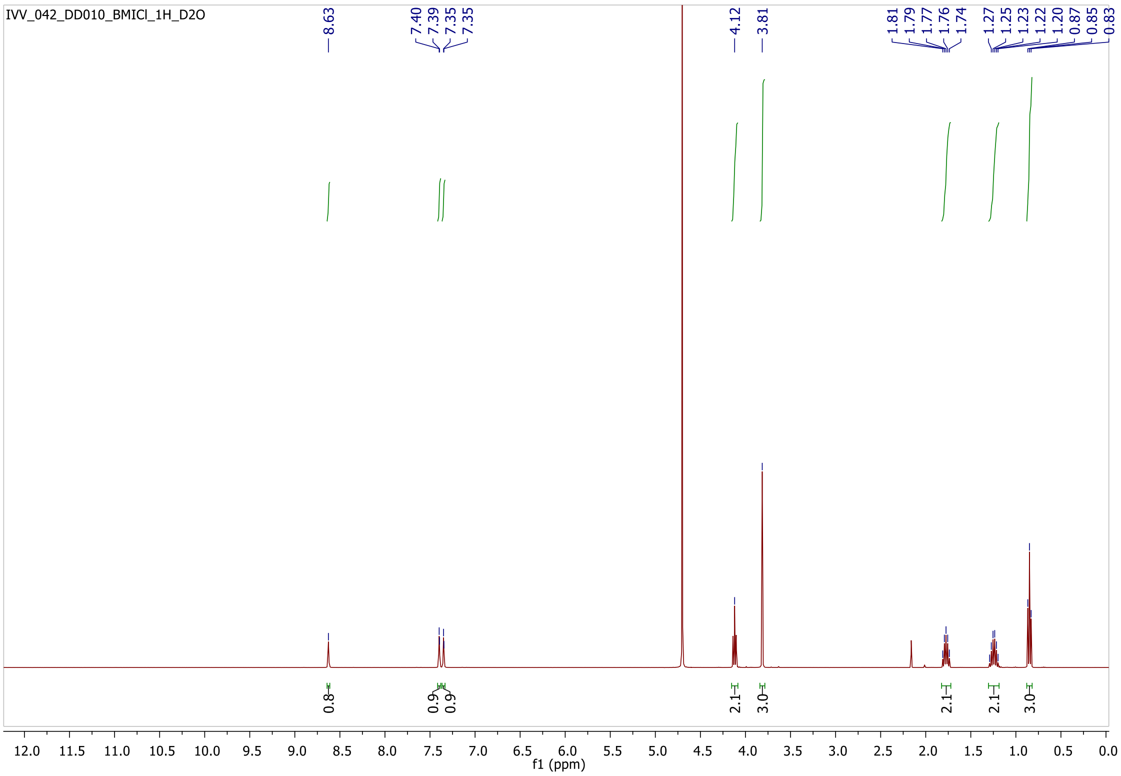

Figure S2. ^{1}HNMR spectra of compound 1.

^{1}H NMR (401 MHz, D_2O) δ 8.63 (s, 1H), 7.40 (d, *J* = 1.7 Hz, 1H), 7.35 (d, *J* = 1.6 Hz, 1H), 4.12 (s, 2H), 3.81 (s, 3H), 1.82 – 1.72 (m, 2H), 1.30 – 1.19 (m, 2H), 0.85 (t, *J* = 7.4 Hz, 3H).

2.1.2. Compound 2: BMI.PIV

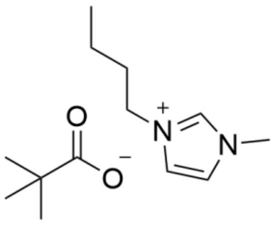

1-butyl-3-methylimidazolium pivalate
Chemical Formula: $C_{13}H_{24}N_2O_2$
Exact Mass: 240,18
Molecular Weight: 240,35
m/z: 240.18 (100.0%), 241.19 (14.4%), 242.19 (1.4%)
Elemental Analysis: C, 64.97; H, 10.07; N, 11.66; O, 13.31

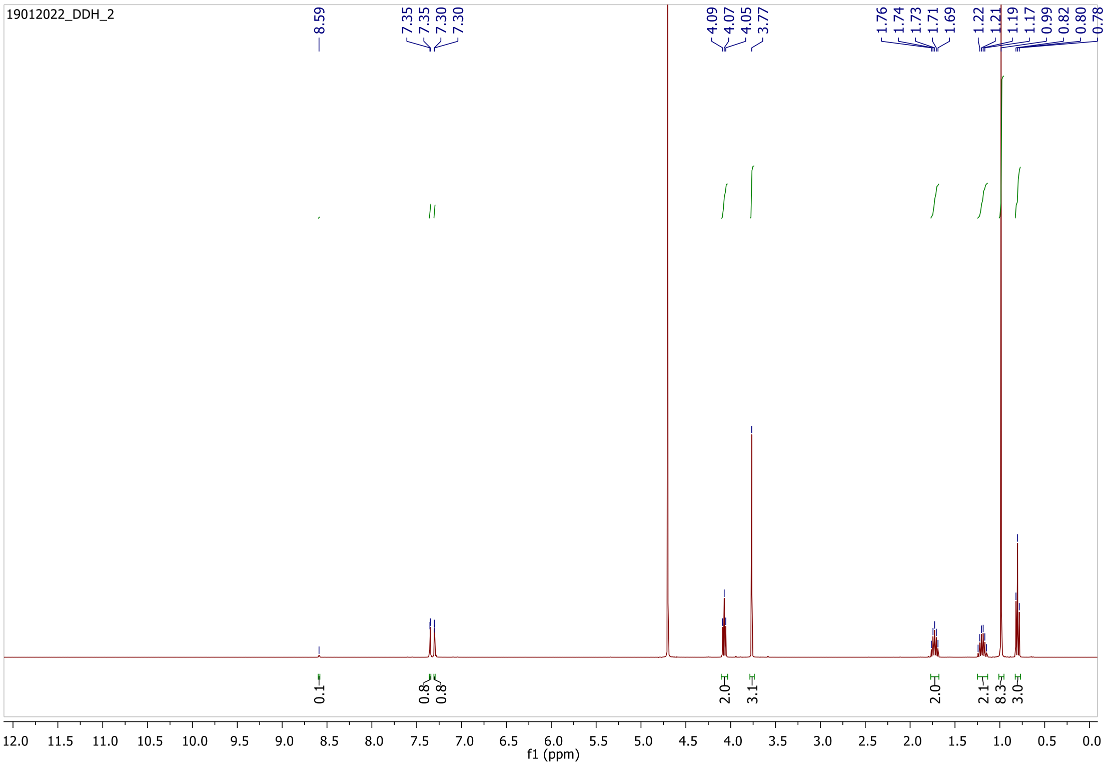

Figure S3. ^{1}HNMR spectra of compound 2.

^{1}H NMR (401 MHz, D_2O) δ 8.59 (s, 1H), 7.35 (d, *J* = 2.0 Hz, 1H), 7.30 (d, *J* = 2.0 Hz, 1H), 4.07 (t, *J* = 7.1 Hz, 2H), 3.77 (s, 3H), 1.73 (dt, *J* = 14.8, 7.4 Hz, 2H), 1.25 – 1.14 (m, 2H), 0.99 (s, 9H), 0.80 (t, *J* = 7.4 Hz, 3H).

2.1.3. Compound 3: BMI.FO

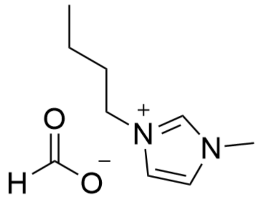

1-butyl-3-methylimidazolium formate
Chemical Formula: $C_9H_{16}N_2O_2$
Exact Mass: 184,12
Molecular Weight: 184,24
m/z: 184.12 (100.0%), 185.12 (10.5%)
Elemental Analysis: C, 58.67; H, 8.75; N, 15.21; O, 17.37

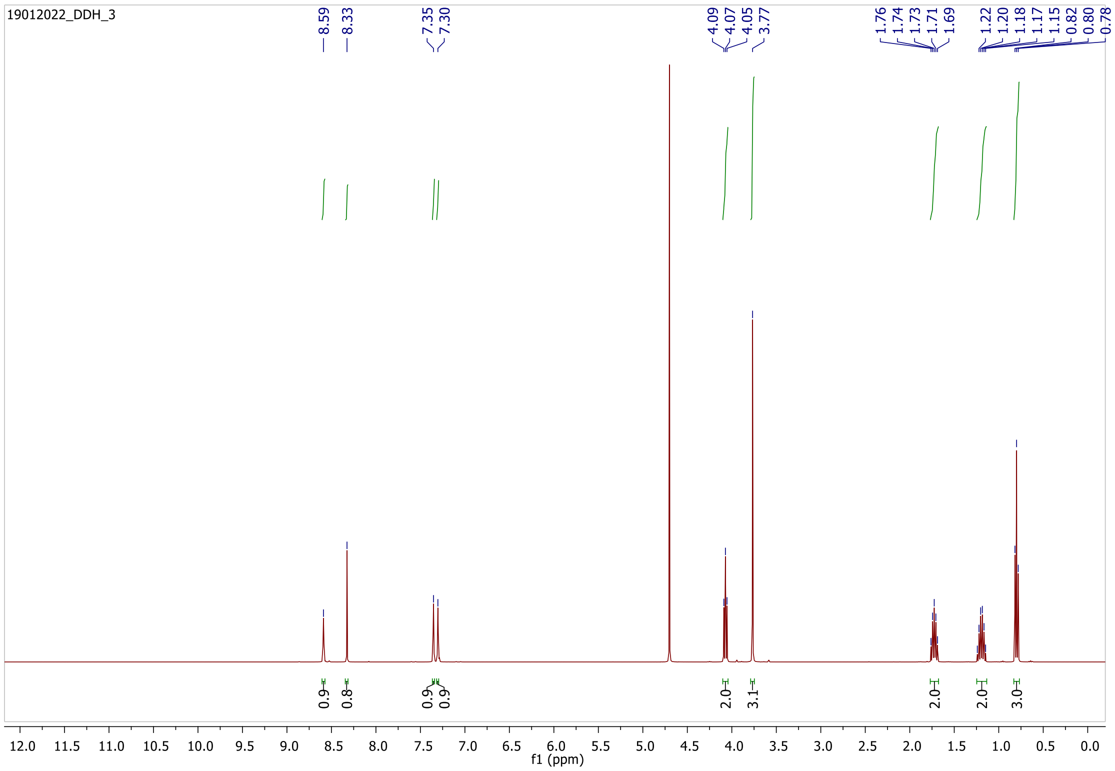

Figure S4. ^{1}HNMR spectra of compound 3.

^{1}H NMR (401 MHz, D_2O) δ 8.59 (s, 1H), 8.33 (s, 1H), 7.35 (t, *J* = 1.8 Hz, 1H), 7.30 (t, *J* = 1.8 Hz, 1H), 4.07 (t, *J* = 7.1 Hz, 2H), 3.77 (s, 3H), 1.72 (dt, *J* = 14.8, 7.4 Hz, 2H), 1.25 – 1.13 (m, 2H), 0.80 (t, *J* = 7.4 Hz, 3H).

2.1.4. Compound 4: BMI.BENZ

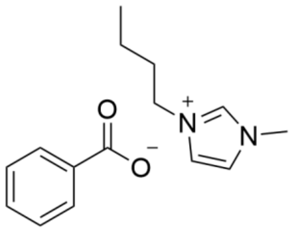

1-butyl-3-methylimidazolium benzoate
Chemical Formula: $C_{15}H_{20}N_2O_2$
Exact Mass: 260,15
Molecular Weight: 260,34
m/z: 260.15 (100.0%), 261.16 (16.5%), 262.16 (1.7%)
Elemental Analysis: C, 69.20; H, 7.74; N, 10.76; O, 12.29

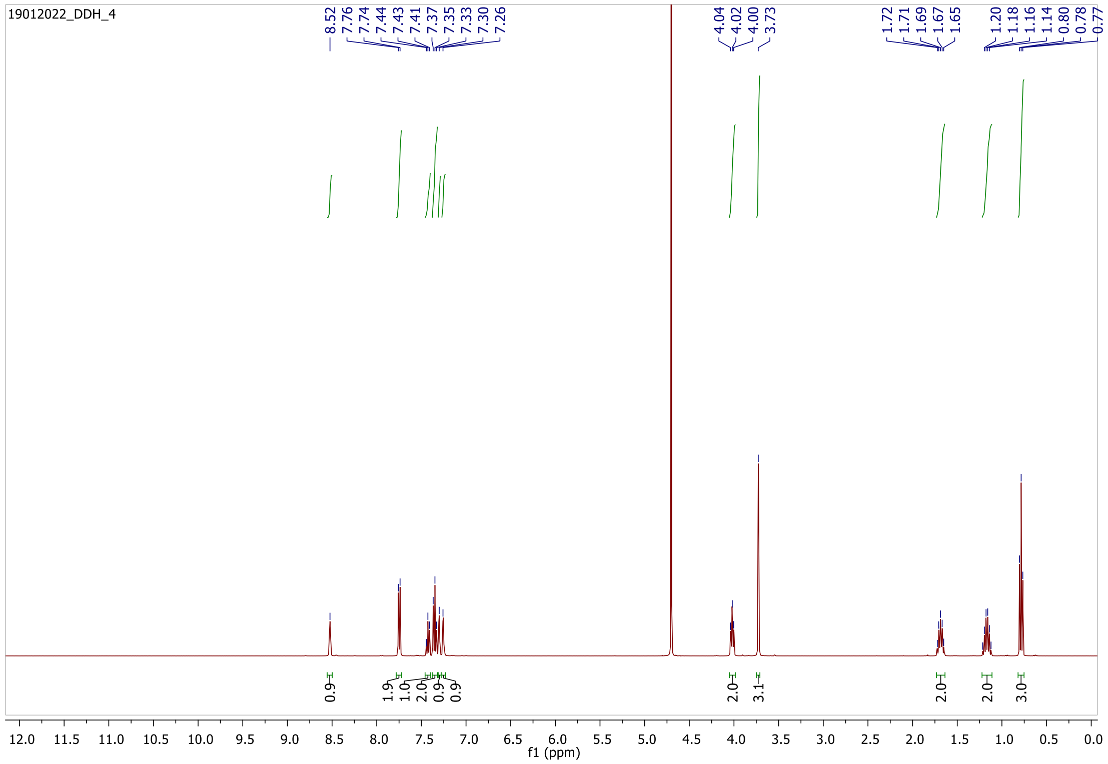

Figure S5. ^{1}HNMR spectra of compound 4.

^{1}H NMR (401 MHz, D_2O) δ 8.52 (s, 1H), 7.75 (d, *J* = 7.3 Hz, 2H), 7.43 (t, *J* = 6.7 Hz, 1H), 7.35 (t, *J* = 7.6 Hz, 2H), 7.30 (d, *J* = 1.5 Hz, 1H), 7.26 (d, *J* = 1.3 Hz, 1H), 4.02 (t, *J* = 7.1 Hz, 2H), 3.73 (s, 3H), 1.69 (p, *J* = 6.6 Hz, 2H), 1.22 – 1.11 (m, 2H), 0.78 (t, *J* = 7.4 Hz, 3H).

2.1.5. Compound 5: BMI.PRO

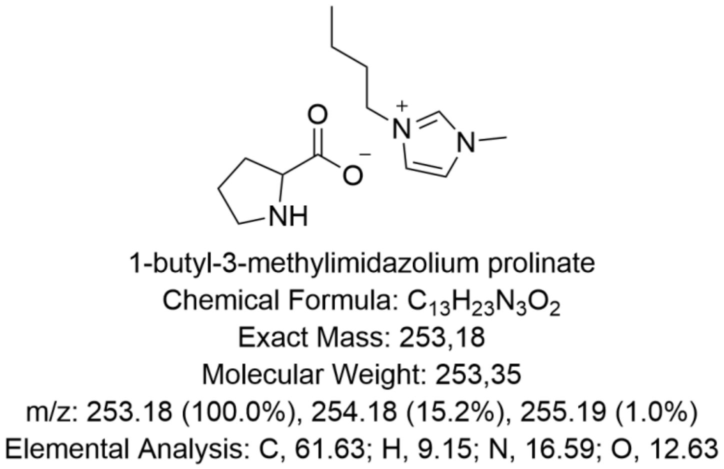

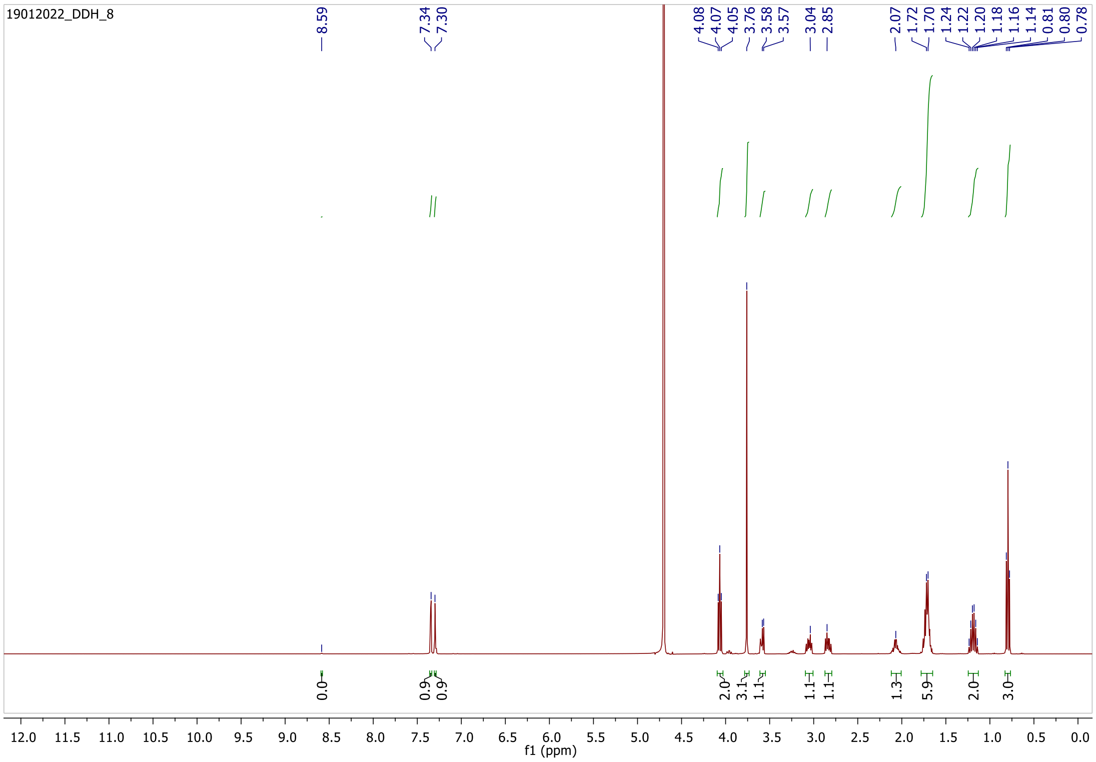

Figure S6. ^{1}HNMR spectra of compound 5.

^{1}H NMR (401 MHz, D_2O) δ 8.59 (s, 1H), 7.35 (d, *J* = 2.0 Hz, 1H), 7.30 (d, *J* = 2.0 Hz, 1H), 4.07 (t, *J* = 7.1 Hz, 2H), 3.76 (s, 3H), 3.61 – 3.55 (m, 1H), 3.09 – 3.01 (m, *J* = 10.9, 6.8 Hz, 1H), 2.87 – 2.79 (m, 1H), 2.12 – 2.01 (m, 1H), 1.78 – 1.65 (m, 6H), 1.25 – 1.13 (m, 2H), 0.80 (t, *J* = 7.4 Hz, 3H).

2.1.6. Compound 6: BMI.MAL

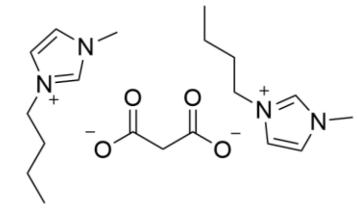

1-butyl-3-methylimidazolium malonate
Chemical Formula: $C_{19}H_{32}N_4O_4$
Exact Mass: 380,24
Molecular Weight: 380,49
m/z: 380.24 (100.0%), 381.25 (21.1%), 382.25 (2.9%), 381.24 (1.5%)
Elemental Analysis: C, 59.98; H, 8.48; N, 14.73; O, 16.82

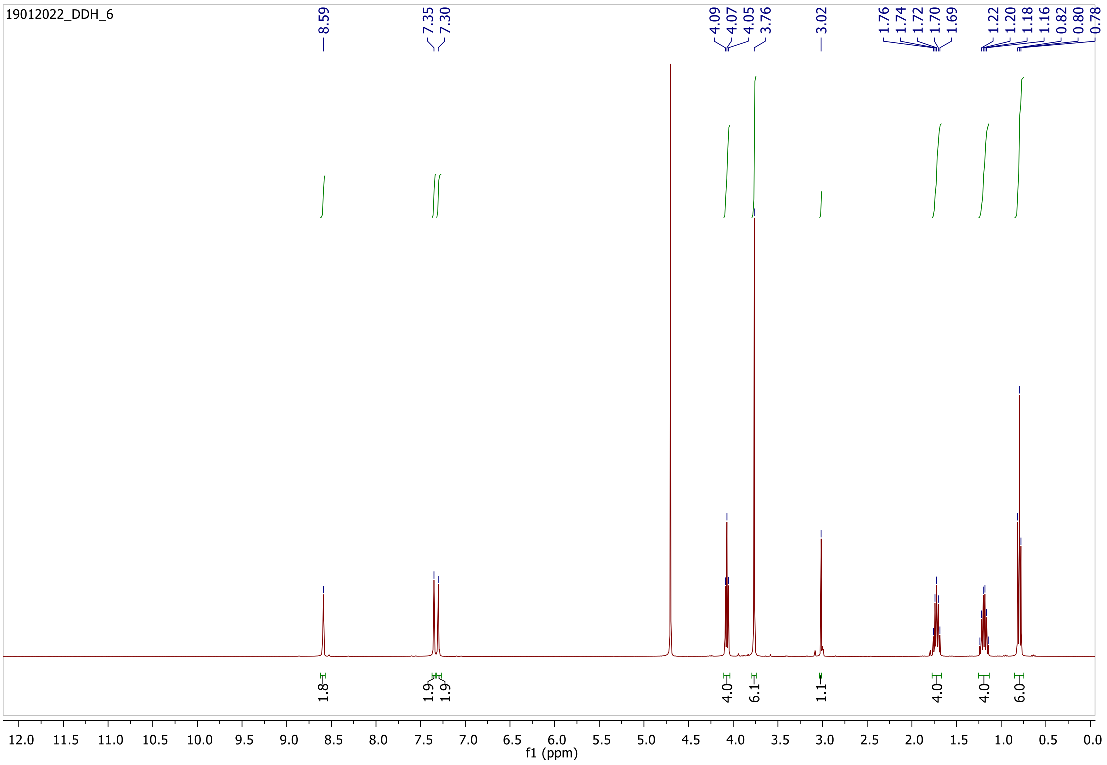

Figure S7. ^{1}HNMR spectra of compound 6.

^{1}H NMR (401 MHz, D_2O) δ 8.59 (s, 2H), 7.35 (t, *J* = 1.8 Hz, 2H), 7.30 (t, *J* = 1.7 Hz, 2H), 4.07 (t, *J* = 7.1 Hz, 4H), 3.76 (s, 6H), 3.02 (s, 1H), 1.72 (dt, *J* = 14.8, 7.3 Hz, 4H), 1.25 – 1.13 (m, 4H), 0.80 (t, *J* = 7.4 Hz, 6H).

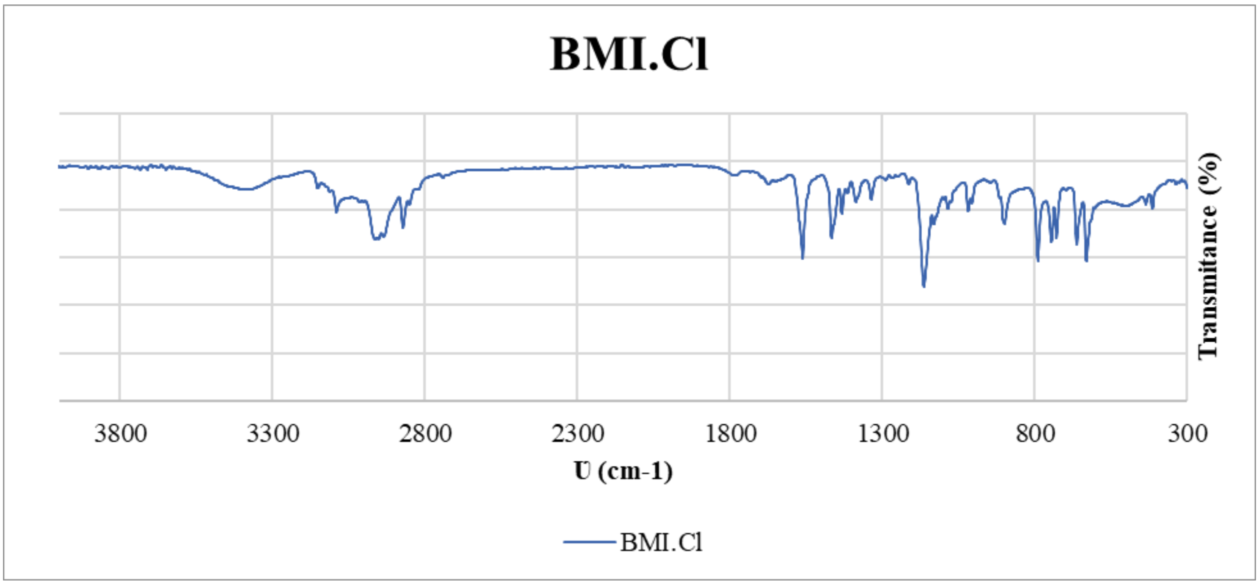

Figure S8. IR spectra of compound 1 (BMI.Cl).

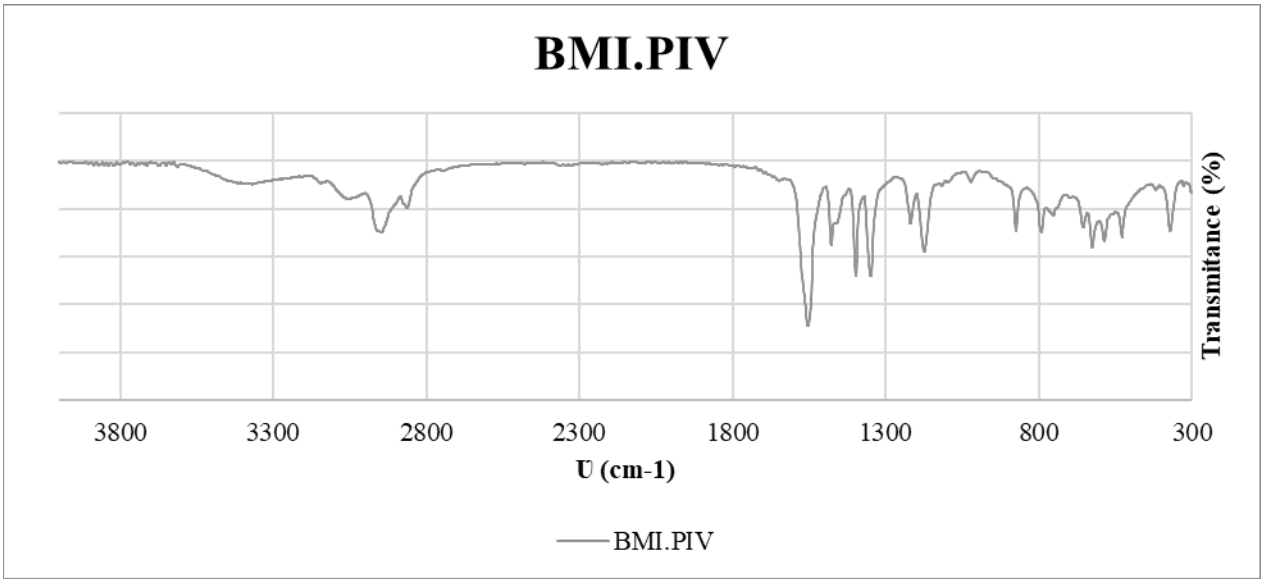

Figure S9. IR spectra of compound 2 (BMI.PIV).

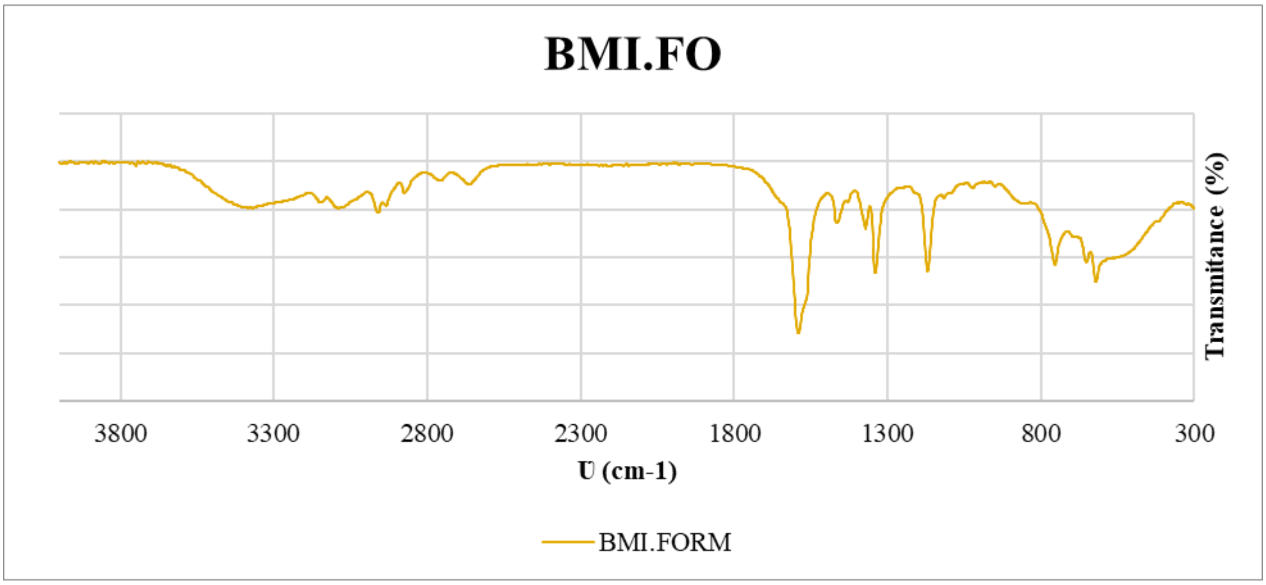

Figure S10. IR spectra of compound 3 (BMI.FORM).

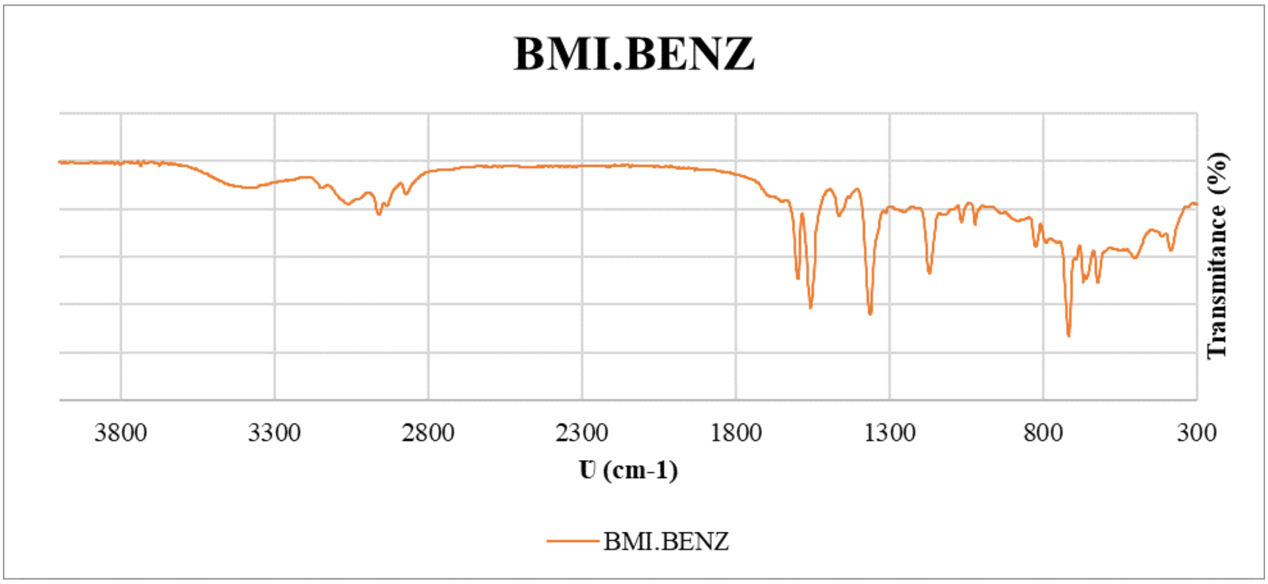

Figure S11. IR spectra of compound 4 (BMI.BENZ).

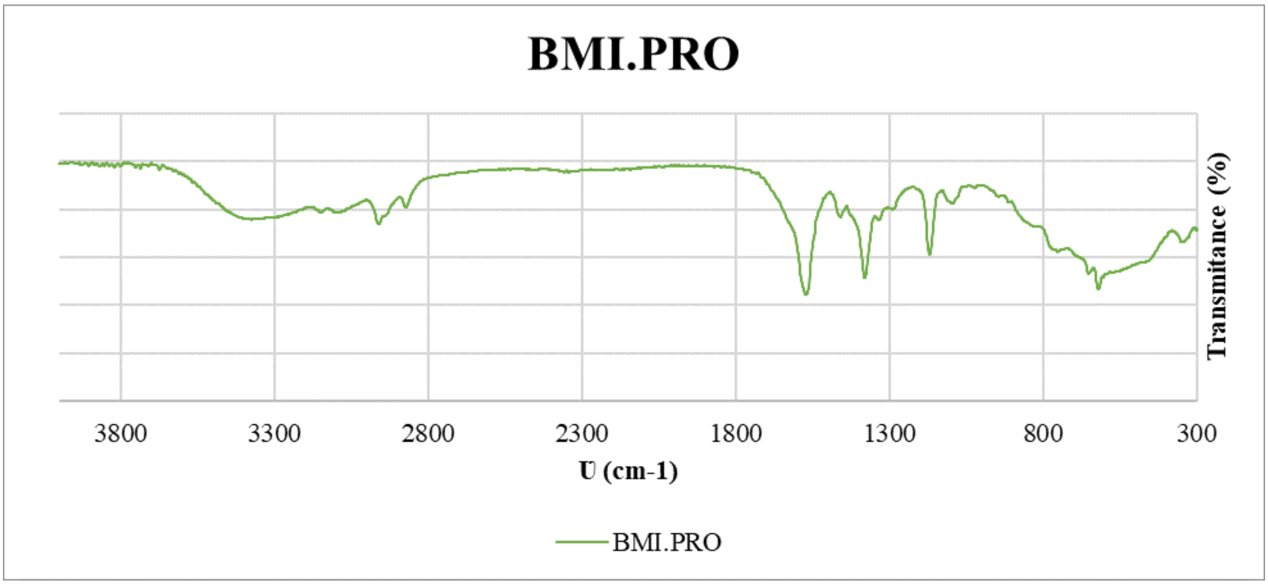

Figure S12. IR spectra of compound 5 (BMI.PRO).

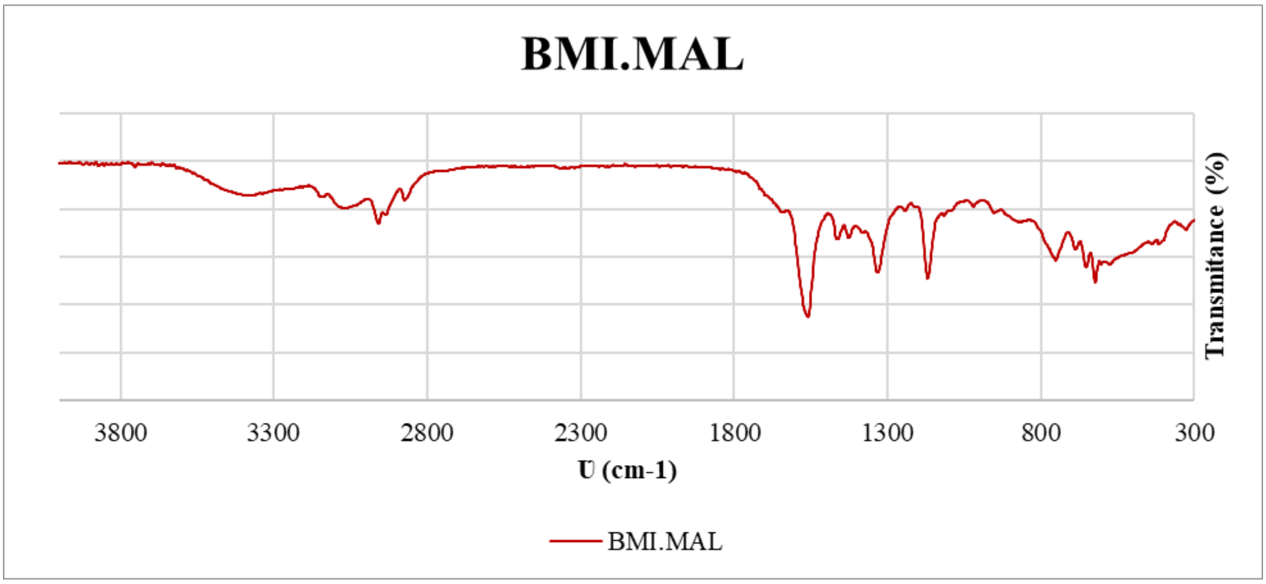

Figure S13. IR spectra of compound 6 (BMI.MAL).

3. Membrane and soaking solutions characterization:

3.1. ATR-IR measurements

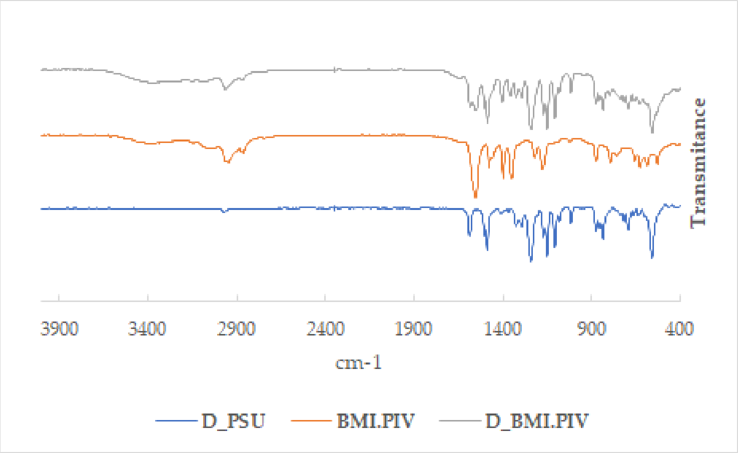

Figure S14. IR stacked spectra D_PSU, BMI.PIV and D_BMI.PIV.

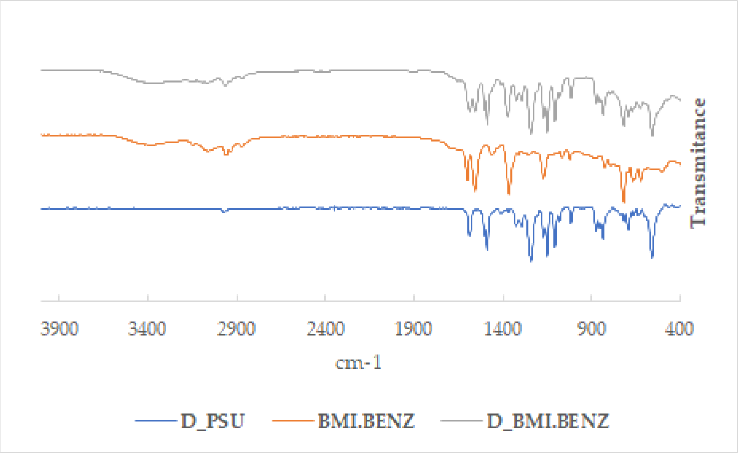

Figure S15. IR stacked spectra D_PSU, BMI.BENZ and D_BMI.BENZ.

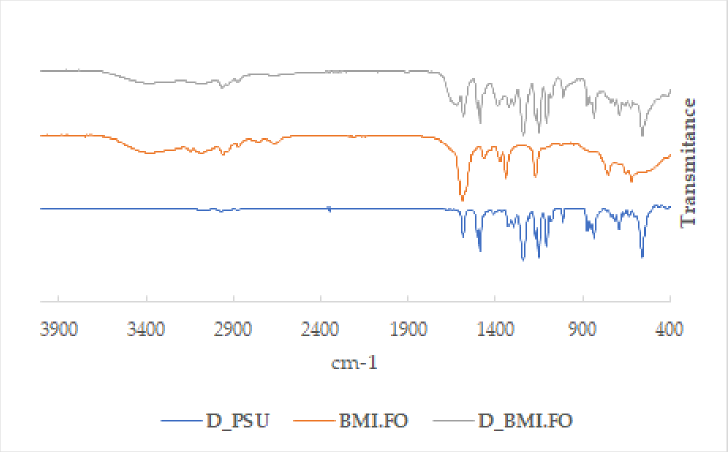

Figure S16. IR stacked spectra D_PSU, BMI.FO and D_BMI.FO.

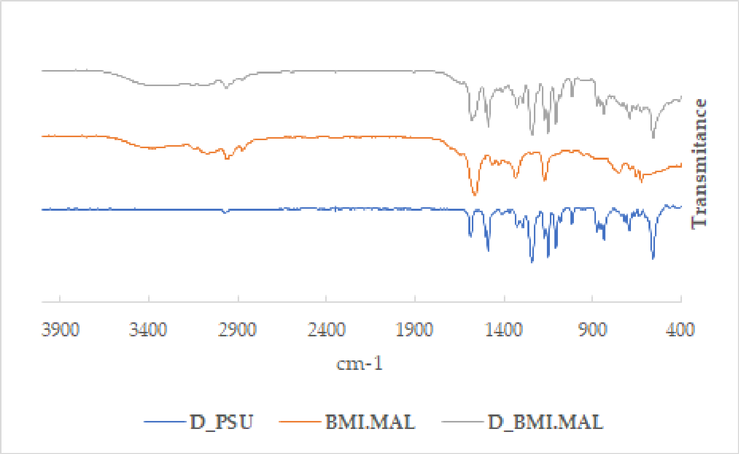

Figure S17. IR stacked spectra D_PSU, BMI.MAL and D_BMI.MAL.

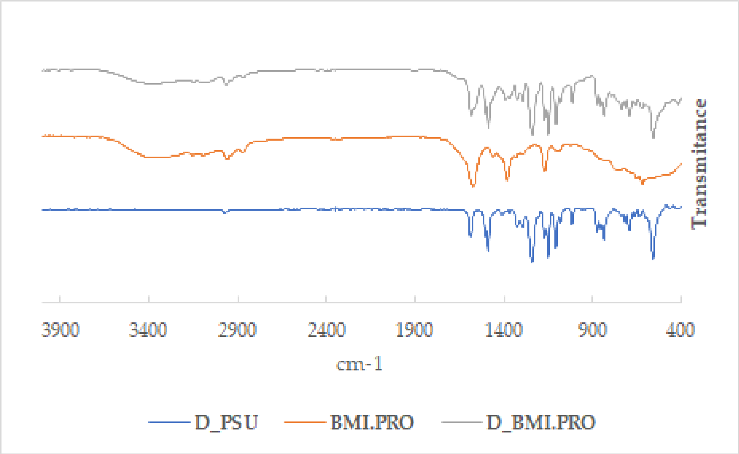

Figure S18. IR stacked spectra D_PSU, BMI.PRO and D_BMI.PRO.

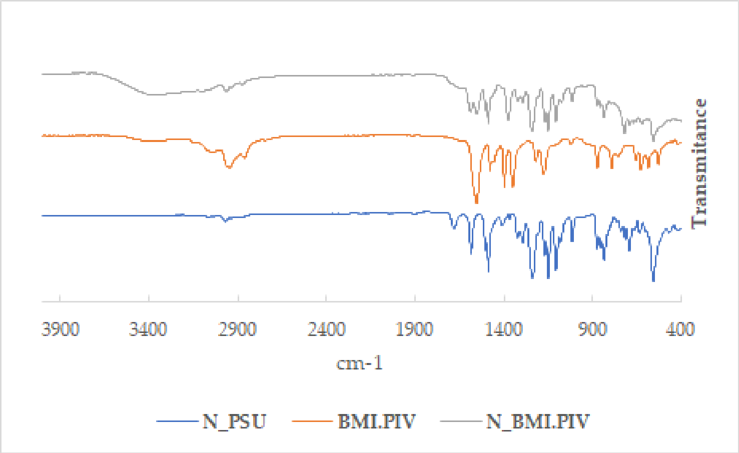

Figure S19. IR stacked spectra N_PSU, BMI.PIV and N_BMI.PIV.

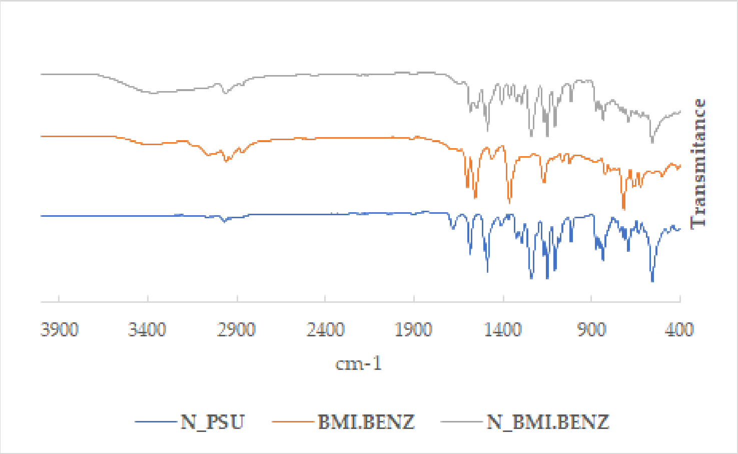

Figure S20. IR stacked spectra N_PSU, BMI.BENZ and N_BMI.BENZ.

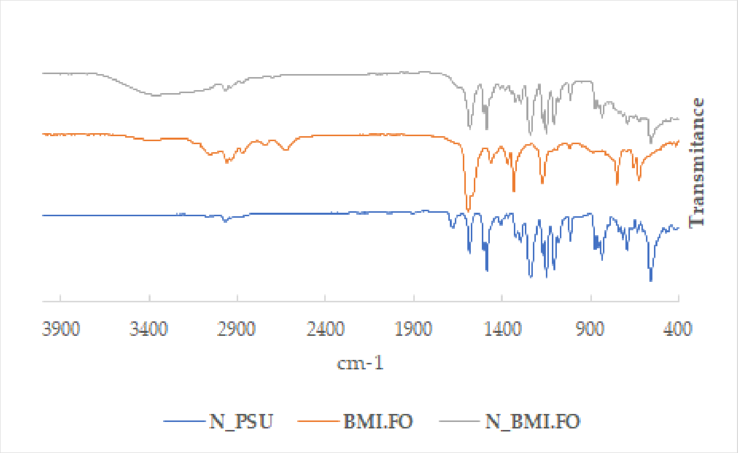

Figure S21. IR stacked spectra N_PSU, BMI.FO and N_BMI.FO.

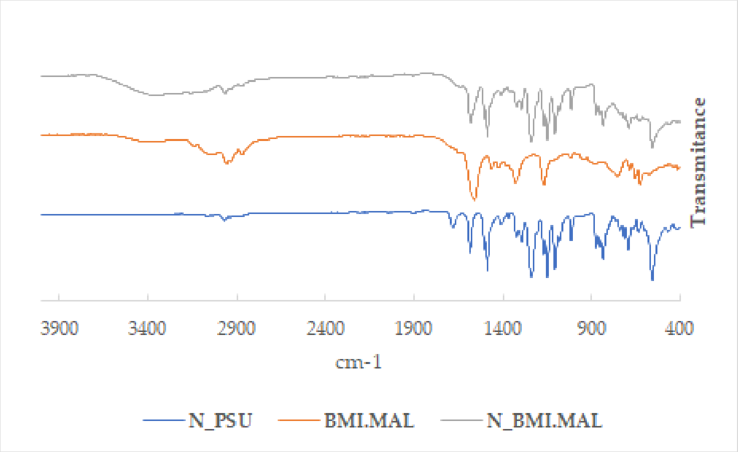

Figure S22. IR stacked spectra N_PSU, BMI.MAL and N_BMI.MAL.

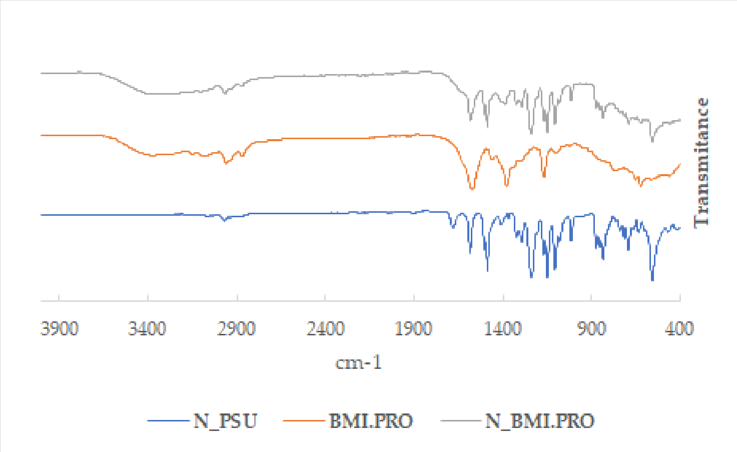

Figure S23. IR stacked spectra N_PSU, BMI.PRO and N_BMI.PRO.

3.2. Elemental Analysis (EDX):

Atom distribution in the membrane:

Carbon (C) Oxygen (O) Chlorine (Cl)

Sulphur (S) Nitrogen (N)

D_PSU

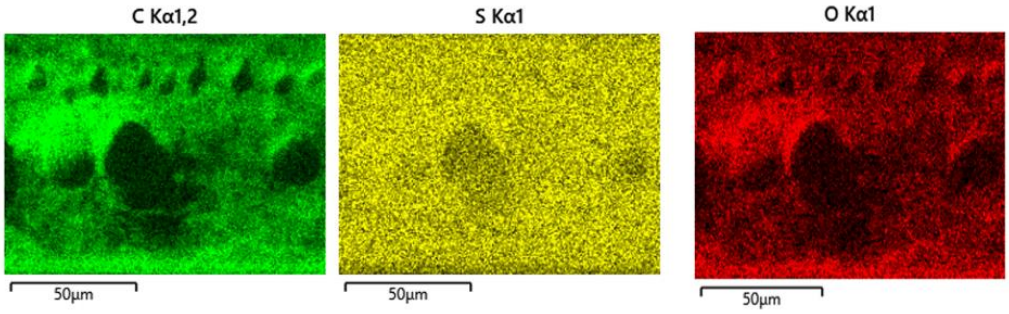

Figure S24. D_PSU membrane EDX.

Table S1. D_PSU weight percentage per element.

Element	Wt (%)
Carbon (C)	77.51
Oxygen (O)	15.84
Sulphur (S)	6.65

D_BMI.PRO

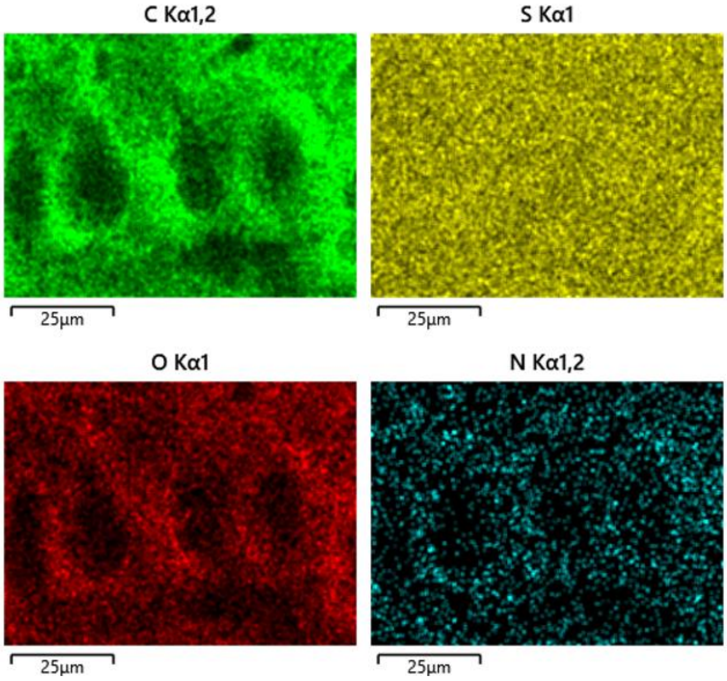

Figure S25. D_BMI.PRO membrane EDX.

Table 2. D_BMI.PRO weight percentage per element.

Element	Wt (%)
Carbon (C)	76.93
Oxygen (O)	16.12
Sulphur (S)	4.9
Nitrogen (N)	2.0

D_BMI.PIV

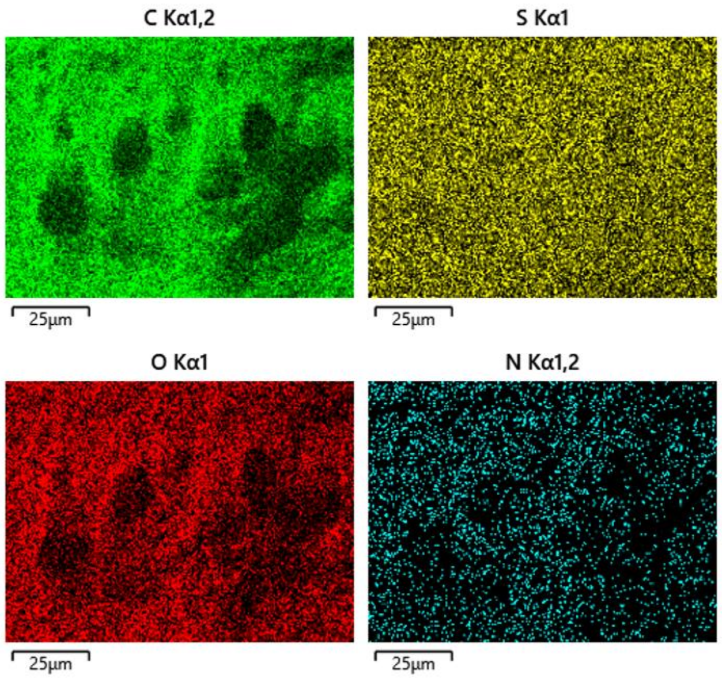

Figure S26. D_BMI.PIV membrane EDX.

Table S3. D_BMI.PIV weight percentage per element.

Element	Wt (%)
Carbon (C)	77.95
Oxygen (O)	18.25
Sulphur (S)	3.9
Nitrogen (N)	2.9

D_BMI.BENZ

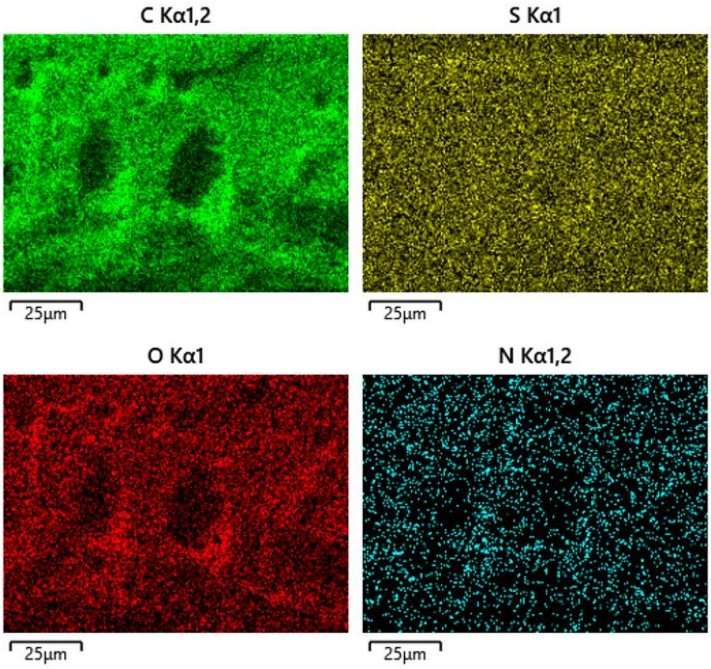

Figure S27. D_BMI.BENZ membrane EDX.

Table S4. D_BMI.BENZ weight percentage per element.

Element	Wt (%)
Carbon (C)	77.27
Oxygen (O)	15.84
Sulphur (S)	4.2
Nitrogen (N)	2.6

D_BMI.FO

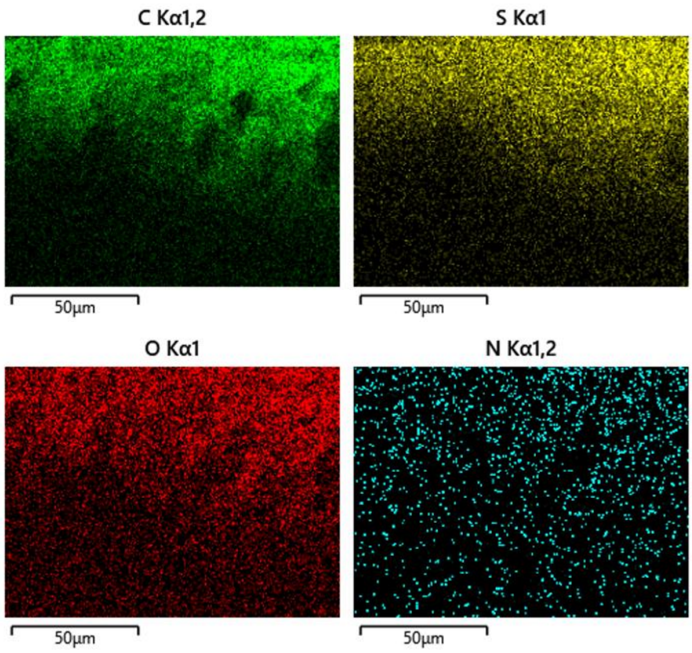

Figure S28. M_5_BMI.FO membrane EDX.

Table S5. D_BMI.FO weight percentage per element.

Element	Wt (%)
Carbon (C)	73.94
Oxygen (O)	19.26
Sulphur (S)	6.2
Nitrogen (N)	0.6

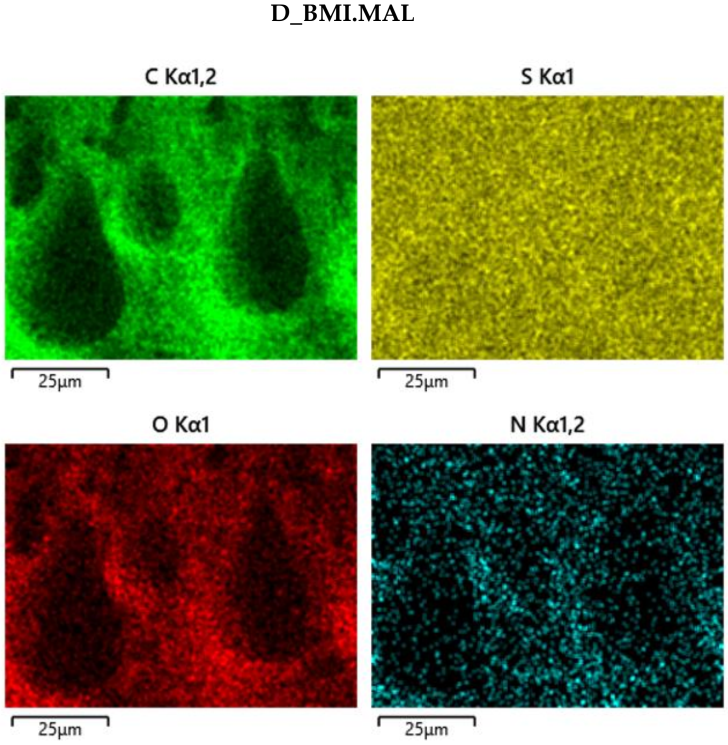

Figure S29. D_BMI.MAL membrane EDX.

Table S6. D_BMI.MAL weight percentage per element.

Element	Wt (%)
Carbon (C)	76.24
Oxygen (O)	16.84
Sulphur (S)	5.8
Nitrogen (N)	1.5

N_PSU

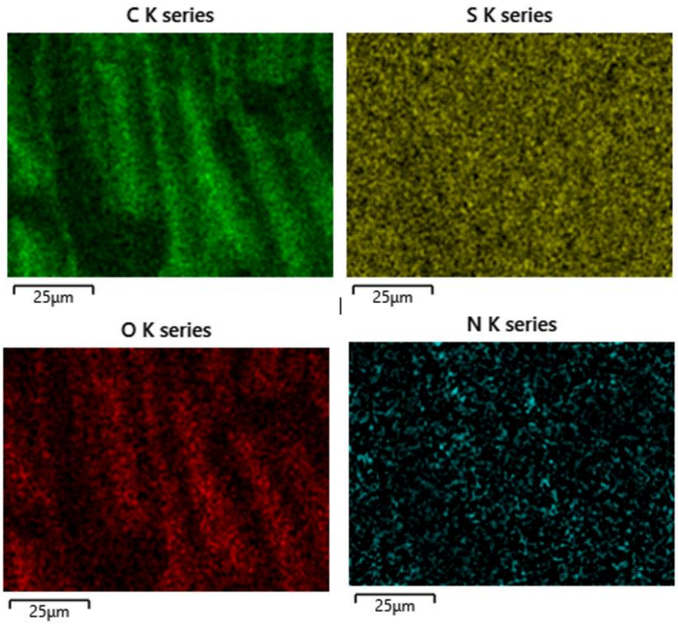

Figure S30. N_PSU membrane EDX.

Table S7. N_PSU weight percentage per element.

Element	Wt (%)
Carbon (C)	76.74
Oxygen (O)	17.48
Sulphur (S)	5.4

N_BMI.PRO

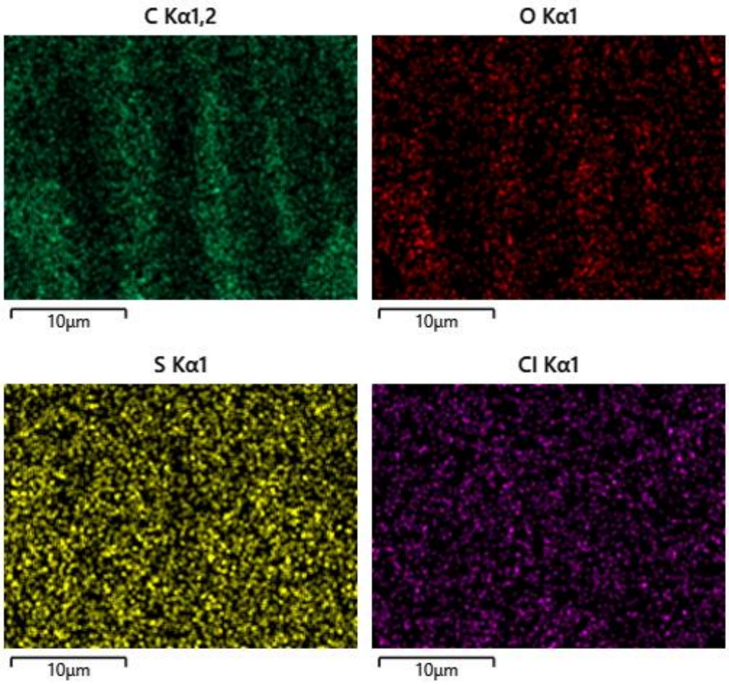

Figure S31. N_BMI.PRO membrane EDX.

Table S8. N_BMI.PRO weight percentage per element.

Element	Wt (%)
Carbon (C)	77.95
Oxygen (O)	16.58
Sulphur (S)	3.94
Nitrogen (N)	Non detected

N_BMI.PIV

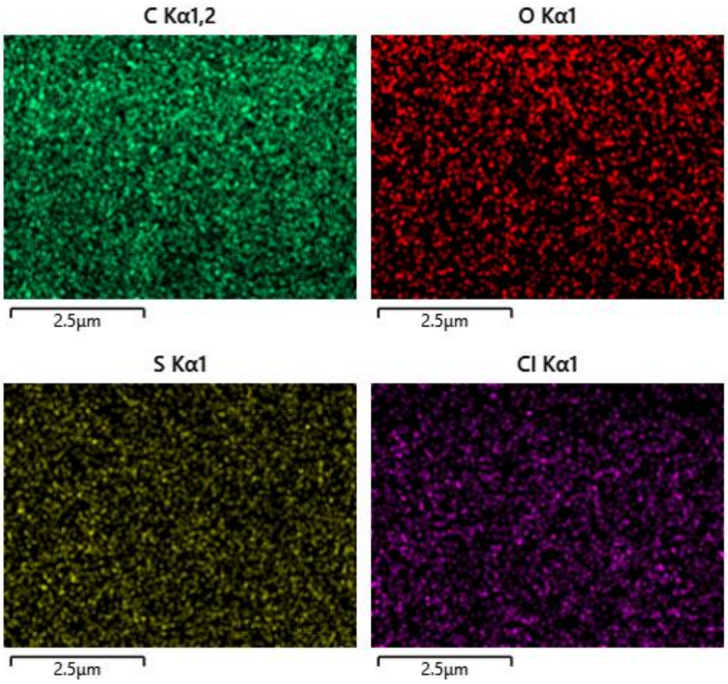

Figure S32. N_BMI.PIV membrane EDX.

Table S9. N_BMI.PIV weight percentage per element.

Element	Wt (%)
Carbon (C)	79.53
Oxygen (O)	16.13
Sulphur (S)	2.66
Nitrogen (N)	Non detected

N_BMI.BENZ

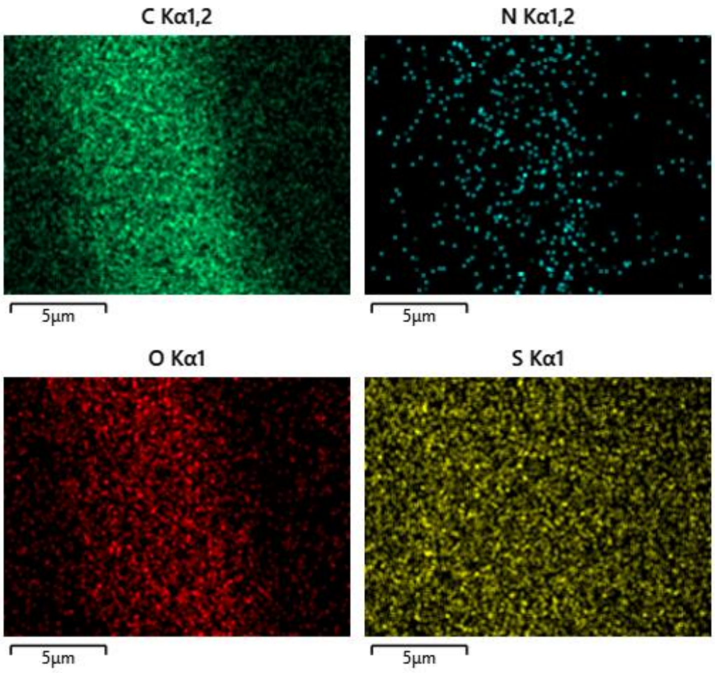

Figure S33. N_BMI.BENZ membrane EDX.

Table S10. N_BMI.BENZ weight percentage per element.

Element	Wt (%)
Carbon (C)	79.29
Oxygen (O)	15.84
Sulphur (S)	3.36
Nitrogen (N)	Non detected

N_BMI.FO

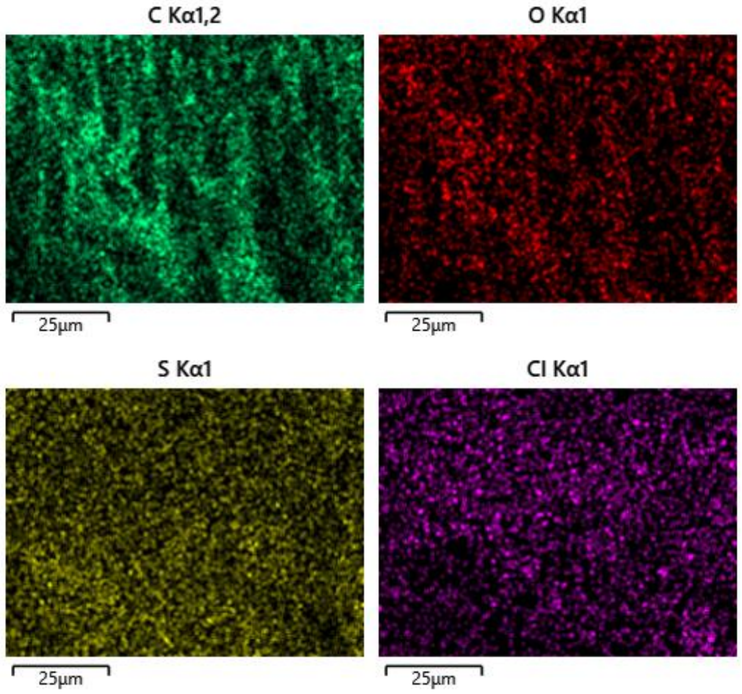

Figure S34. N_BMI.FO membrane EDX.

Table S11. N_BMI.FO weight percentage per element.

Element	Wt (%)
Carbon (C)	76.84
Oxygen (O)	17.15
Sulphur (S)	4.49
Nitrogen (N)	Non detected

N_BMI.MAL

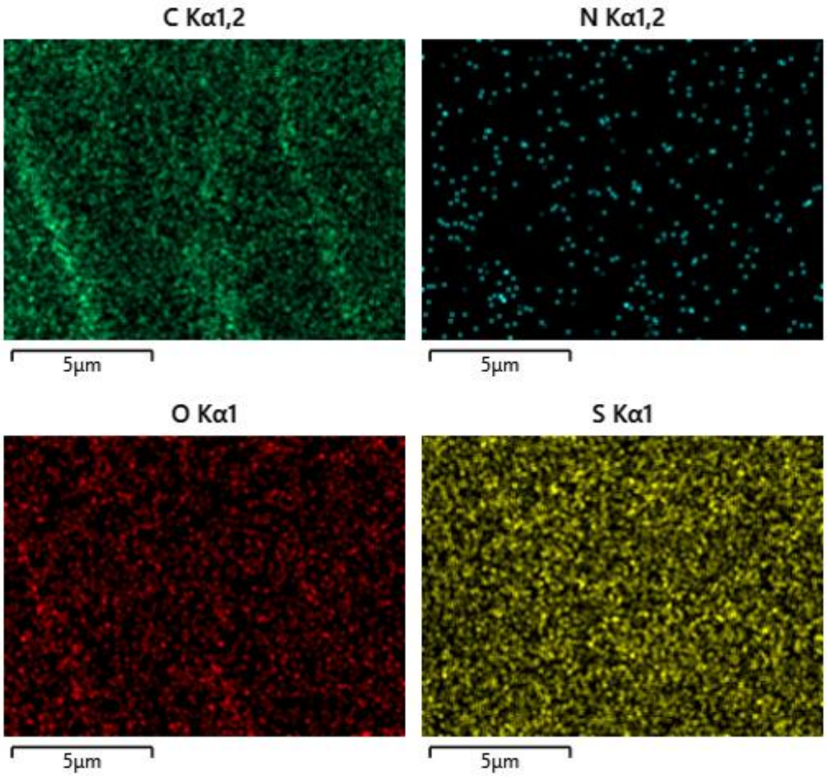

Figure S35. N_BMI.MAL membrane EDX.

Table S12. N_BMI.MAL weight percentage per element.

Element	Wt (%)
Carbon (C)	75.90
Oxygen (O)	17.07
Sulphur (S)	4.87
Nitrogen (N)	Non detected

3.3. IL's density:

Table S13. Ionic liquids density and 75% (w/w) solution density.

Compound	Name	Density (g/mL)	Density solution 75% (w/w) in miliQ water (g/mL)
1	BMI.Cl	1.086[1]	1.0481
2	BMI.PIV	1.0832	1.0745
3	BMI.FORM	1.0757	1.0407
4	BMI.BENZ	1,1040	1,1010
5	BMI.PRO	1,1354	1,1469
6	BMI.MAL	1.1345	1.0588

[1] On line source: https://www.scbt.com/es/p/1-butyl-3-methylimidazolium-chloride-79917-90-1 (23/05/2022)

3.4. IL's 75% (w/w) solution viscosity:

Table S14. Ionic liquids 75% (w/w) solution viscosity.

Compound	Name	Viscosity solution 75% in miliQ water (mPa*s)
1	BMI.Cl	21.30 ± 0.1
2	BMI.PIV	27.1 ± 0.8
3	BMI.FORM	11.9 ± 0.8
4	BMI.BENZ	25.7 ± 0.3
5	BMI.PRO	39.8 ± 0.9
6	BMI.MAL	31 ± 1

3.5. IL's 75% (w/w) solution surface tension:

Table S15. Ionic liquids 75% (w/w) solution surface tension.

Compound	Name	Surface tension solution 75% in miliq water (mN/m)
---	Mili Q H_2o	82 ± 2
1	BMI.Cl	54.9 ± 0.6
2	BMI.PIV	62 ± 1
3	BMI.FORM	52 ± 3
4	BMI.BENZ	48 ± 4
5	BMI.PRO	57 ± 1
6	BMI.MAL	62 ± 4

3.6. Contact Angle (CA)

- DMF Membranes

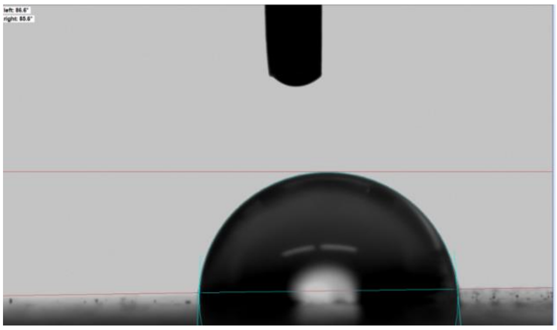

Figure S36. D_Blank top surface CA with mili Q H_2O.

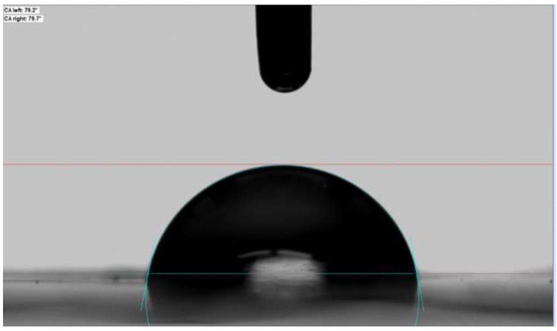

Figure S37. D_Blank bottom surface CA with mili Q H_2O.

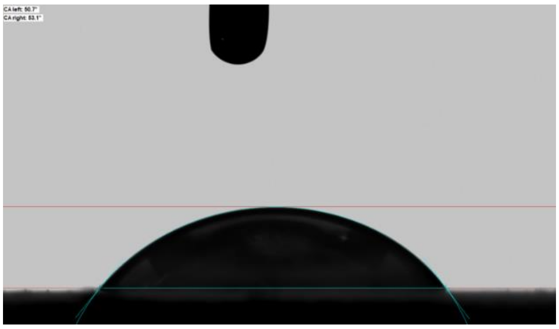

Figure S38. D_Blank top surface CA with BMI.PRO.

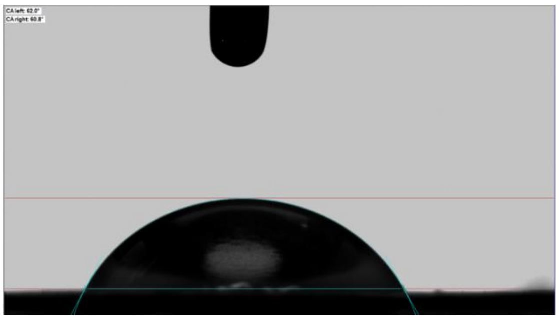

Figure S39. D_Blank bottom surface CA with BMI.PRO.

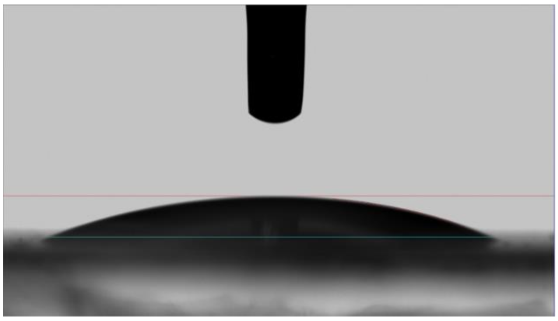

Figure S40. D_Blank top surface CA with BMI.PIV.

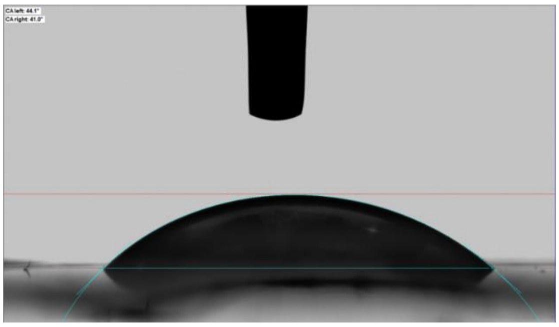

Figure S41. D_Blank bottom surface CA with BMI.PIV.

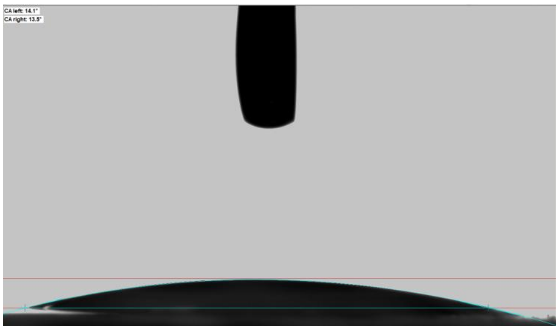

Figure S42. D_Blank top surface CA with BMI.BENZ.

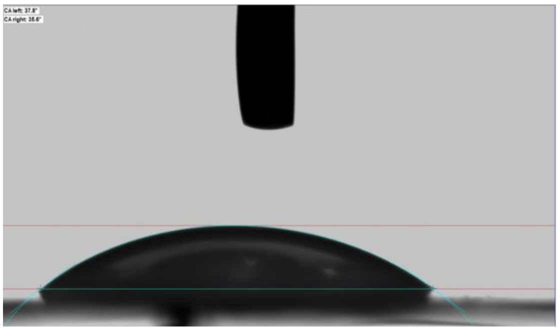

Figure S43. D_Blank bottom surface CA with BMI.BENZ.

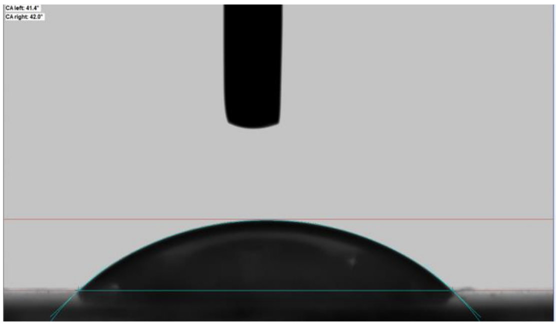

Figure S44. D_Blank top surface CA with BMI.FO.

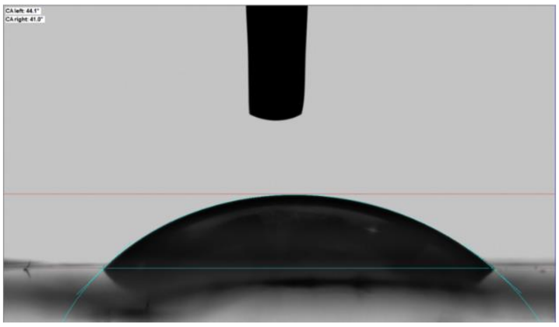

Figure S45. D_Blank bottom surface CA with BMI.FO.

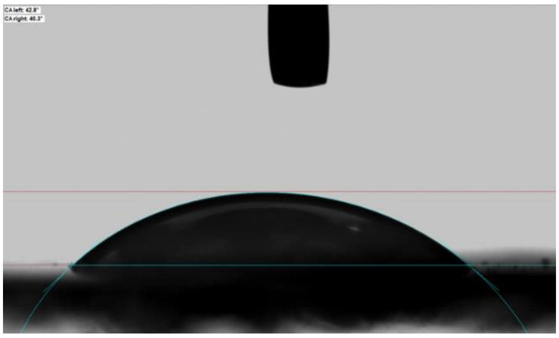

Figure S46. D_Blank top surface CA with BMI.MAL.

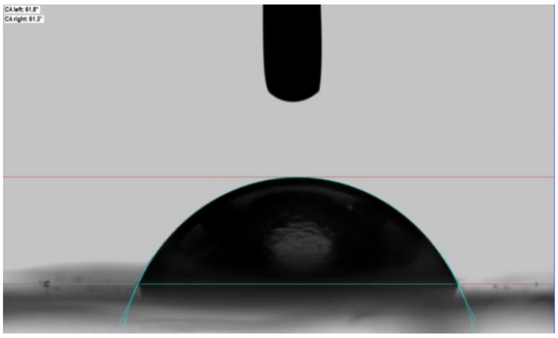

Figure S47. D_Blank bottom surface CA with BMI.MAL.

- NMP membranes

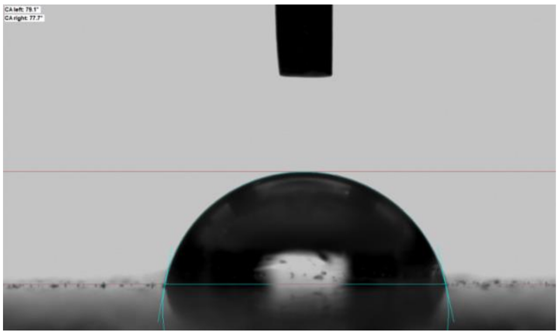

Figure S48. N_Blank top surface CA with mili Q H_2O.

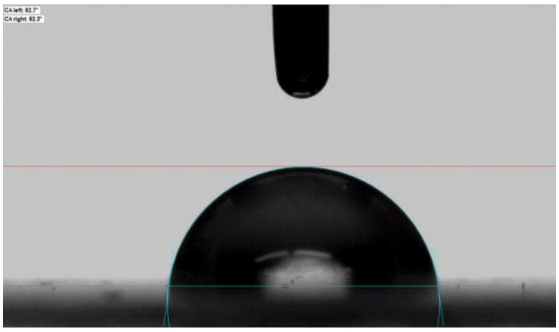

Figure S49. N_Blank bottom surface CA with mili Q H_2O.

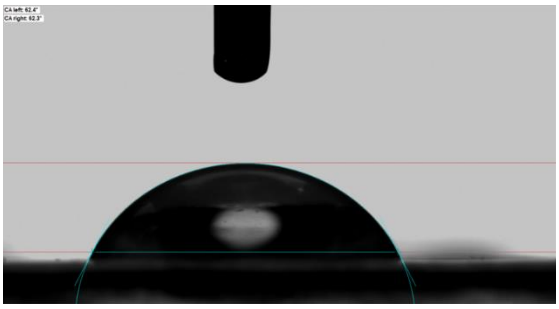

Figure S50. N_Blank top surface CA with BMI.PRO.

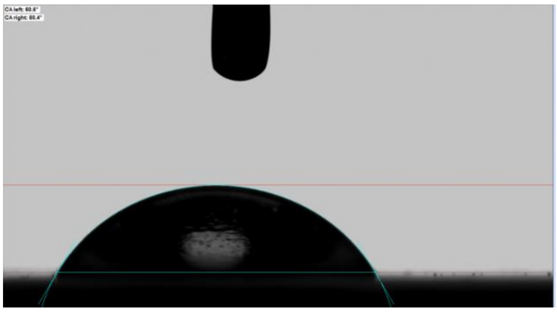

Figure S51. N_Blank bottom surface CA with BMI.PRO.

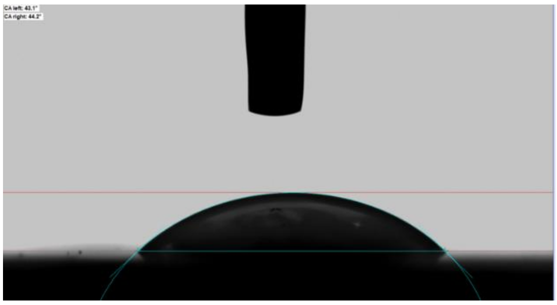

Figure S52. N_Blank top surface CA with BMI.PIV.

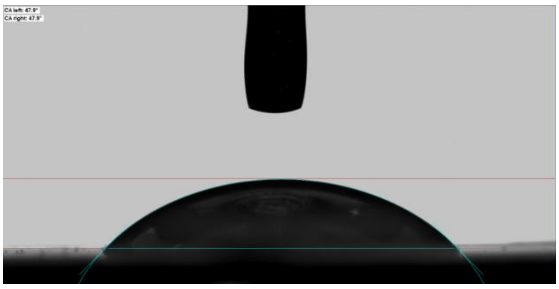

Figure S53. N_Blank bottom surface CA with BMI.PIV.

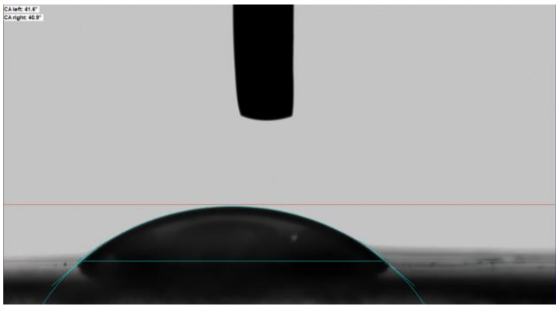

Figure S54. N_Blank top surface CA with BMI.BENZ.

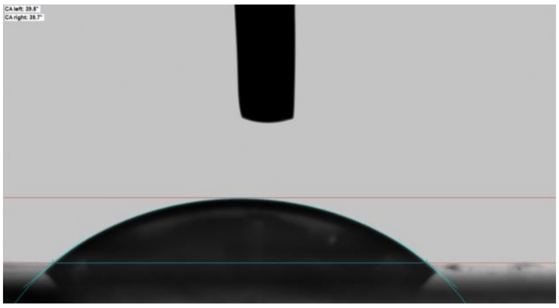

Figure S55. N_Blank bottom surface CA with BMI.BENZ.

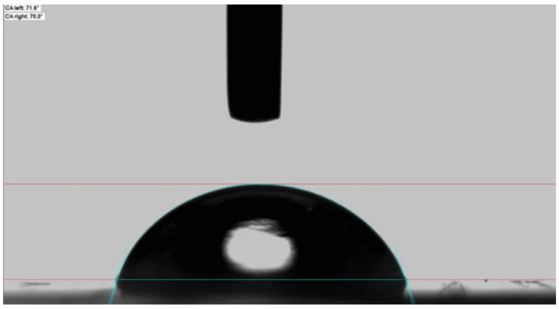

Figure S56. N_Blank top surface CA with BMI.FO.

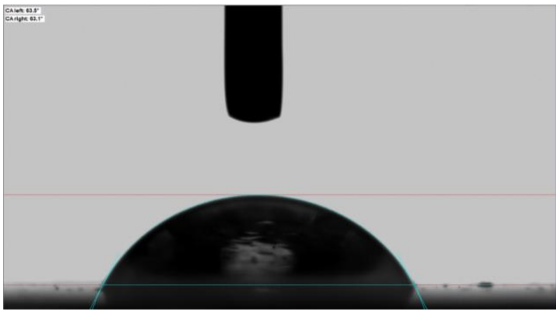

Figure S57. N_Blank bottom surface CA with BMI.FO.

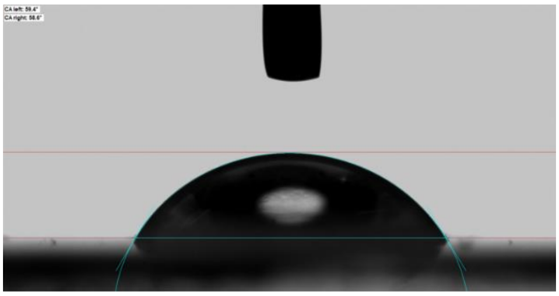

Figure S58. N_Blank top surface CA with BMI.MAL.

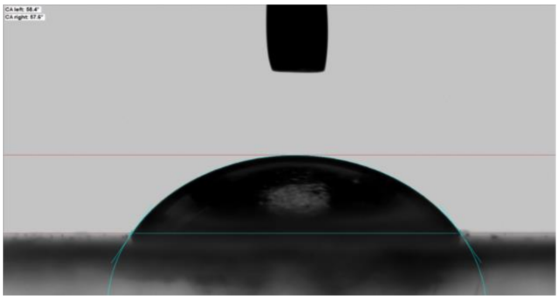

Figure S59. N_Blank bottom surface CA with BMI.MAL.

4. Membrane solubility testing

4.1. Solubility calculations:

$$n_{CO_2} = \frac{(P_i - P_f) \times (V_v - V_m)}{R \times T} \quad (1)$$

In which:
n_{CO2} – adsorbed moles [mol]
P_i – initial pressure [Pa]
P_f – final pressure [Pa]
V_v – volume empty chamber [m^3]
V_m – volume membrane material [m^3]
R – gas constant (8,314 [$m^3 \cdot Pa \cdot K^{-1} \cdot mol^{-1}$])
T – temperature [k]

$$S = \frac{V_{stp}}{V_m \times P_f} \quad (2)$$

In which:
S – solubility coefficient [m^3 (STP) $m^{-3}_{membrane}$ atm^{-1}]
V_{stp} – the volume of CO_2 adsorbed in standard conditions STP [m^3] (at 1 atm and 273.15 K)
V_m – membrane material volume [m^3]
P_f – final pressure after stabilization [atm]

Normalized values of the amount of CO_2 solubilized in the ionic liquids are obtained by subtracting the CO_2 absorbed in the blank membranes.

4.2. Solubility results:

Table S16. Solubilities for NMP membranes.

Membrane	CO_2 mol/g of membrane	Solubility coefficient (S)(m^3(STP) $m^{-3}_{membrane}$ atm^{-1})	Solubility coefficient normalized by g of IL	CO_2 solubility of ILs (molCO_2/mol IL)
N_PSU_1	2.811 x 10^{-4}	1.83 x 10^{5}	0	
N_BMI.PRO_1	3.503 x 10^{-4}	2.52 x 10^{6}	1.908 x 10^{6}	0.17
N_BMI.PRO_2	2.866 x 10^{-4}	9.52 x 10^{5}	5.436 x 10^{5}	0.15
N_BMI.PIV_1	4.50 x 10^{-4}	1.64 x 10^{7}	1.404 x 10^{7}	0.16
N_BMI.PIV_2	3.88 x 10^{-4}	7.30 x 10^{11}	5.973 x 10^{11}	0.14
N_BMI.BENZ_1	1.373 x 10^{-4}	2.42 x 10^{11}	1.238 x 10^{11}	0.05
N_BMI.BENZ_2	1.911 x 10^{-4}	3.40 x 10^{11}	1.957 x 10^{11}	0.07
N_BMI.FO_1	9.58 x 10^{-5}	5.08 x 10^{5}	--	0.03
N_BMI.FO_2	1.24 x 10^{-4}	3.04 x 10^{5}	--	0.05
N_BMI.MAL_1	2.63 x 10^{-4}	4.51 x 10^{5}	1.985 x 10^{5}	0.24
N_BMI.MAL_2	1.910 x 10^{-4}	1.05 x 10^{6}	4.991 x 10^{5}	0.12

Table S17. Solubilities for DMF membranes.

Membrane	CO_2 mol/g of membrane	Solubility coefficient (S)(m^3(STP) $m^{-3}{}_{membrane}$ atm^{-1})	Solubility coefficient normalized by g of IL	CO_2 solubility of ILs (molCO_2/mol IL)
D_PSU_1	6.286×10^{-5}	4.16×10^{4}	0	
D_BMI.PRO_1	--	--	--	--
D_BMI.PRO_2	7.731×10^{-5}	7.27×10^{4}	1.676×10^{4}	0.07
D_BMI.PIV_1	4.31×10^{-4}	5.25×10^{6}	4.967×10^{6}	0.15
D_BMI.PIV_2	2.12×10^{-4}	1.71×10^{6}	1.494×10^{6}	0.07
D_BMI.BENZ_1	8.03×10^{-5}	2.72×10^{6}	1.853×10^{6}	0.03
D_BMI.BENZ_2	5.06×10^{-5}	5.48×10^{5}	2.938×10^{5}	0.02
D_BMI.FO_1	1.27×10^{-4}	1.36×10^{5}	7.524×10^{4}	0.08
D_BMI.FO_2	2.12×10^{-4}	2.03×10^{5}	1.512×10^{5}	0.15
D_BMI.MAL_1	1.54×10^{-4}	1.31×10^{5}	8.235×10^{4}	0.23
D_BMI.MAL_2	1.33×10^{-4}	9.95×10^{4}	6.307×10^{4}	0.22

Table S18. Solubility in pure ILs.

Pure IL	CO_2 mol/g of IL	Solubility coefficient normalized by g of IL	CO_2 solubility of ILs (molCO2/mol IL)
BMI.PRO_1	6.46×10^{-5}	3.833×10^{6}	0.016
BMI.PIV_1	3.48×10^{-4}	2.293×10^{7}	0.083
BMI.BENZ_1	1.35×10^{-4}	8.099×10^{6}	0.035
BMI.FO_1	3.28×10^{-5}	2.152×10^{7}	0.060
BMI.MAL_1	2.12×10^{-4}	1.290×10^{7}	0.080
